# Biocompatibility and biofilm formation on conventional and CAD/CAM provisional implant restorations

**DOI:** 10.1186/s12903-023-03468-z

**Published:** 2023-10-05

**Authors:** Tipparat Parakaw, Nisarat Ruangsawasdi, Pornpen Dararat, Chareerut Phruksaniyom, Sirada Srihirun, Pobploy Petchmedyai

**Affiliations:** 1 Department of Pharmacology, Faculty of Dentistry, Mahidol University, Bangkok, Thailand; 2 Dental Implant Center, Faculty of Dentistry, Mahidol University, Bangkok, Thailand

**Keywords:** Dental prosthesis, Computer-aided manufacturing, Porphyromonas gingivalis, Fibroblast, Cytotoxicity tests

## Abstract

Dental implant treatment is a complex and sophisticated process, and implant provisional restorations play a vital role in ensuring its success. The advent of computer-aided design and computer-aided manufacturing (CAD/CAM) technology has revolutionized the field of implant restorations by providing improved precision leading to a reduction in chair time and more predictable treatment outcomes. This technology offers a promising solution to the drawbacks of conventional methods and has the potential to transform the way implant procedures are approached. Despite the clear advantages of CAD/CAM over conventional provisional implant restorations including higher accuracy of fit and superior mechanical properties, little research has been conducted on the biological aspect of these novel restorations. This study aims to fill that gap, comprehensively assessing the biocompatibility, gingival tissue attachment and biofilm formation of a range of provisional implant restorations using CAD/CAM technology through milling and 3-D printing processes compared to conventional fabrication. The biocompatibility of the tested restorations was assessed by MTT assay, Calcein-AM assay as well as SEM analysis. The surface roughness of the tested samples was evaluated, alongside the attachment of Human Gingival Fibroblasts (HGF) cells as well as biofilm formation, and estimated *Porphyromonas gingivalis* (*P. gingivalis*) cell count from DNA detection.

The results showed all tested provisional implant restorations were non-toxic and good HGF cell attachment but differed in their quantity of biofilm formation, with surface texture influenced by the material and fabrication technique, playing a role. Within the limitation of this study, the findings suggest that CAD/CAM-fabricated provisional implant restorations using a milling technique may be the most favourable among tested groups in terms of biocompatibility and periodontal-related biofilm formation.

## Background

Provisional restorations play a crucial role in the success of implant therapy, as they help to preserve and shape peri-implant soft tissue prior the delivery of definitive restoration [1–5]. A range of materials is currently available for the fabrication of provisional implant restorations, including polymethylmethacrylate (PMMA)-based materials and bis-acryl- based materials. PMMA self-cured acrylic resin is a material that is widely acknowledged for its versatility, affordability, and ease of handling. Its rapid setting time enables efficient chairside production of provisional restorations. While bis-acryl-based materials have become increasingly widely used due to their low polymerization shrinkage and high esthetics, allowing for precise replication of the tooth anatomy and color matching. These materials offer enhanced stability during the healing phase, which is vital for successful implant therapy. Both PMMA and bis-acryl provisional restorations have been extensively researched and demonstrated favorable clinical outcomes, making them the standard materials in clinical implant practice [6, 7]. In recent years, with the advent of computer-aided design and computer-aided manufacturing (CAD/CAM) technology, the fabrication of implant restorations has undergone a digital transformation, enabling the design of provisional restorations using computer software and their subsequent production through milling or 3-D printing machines. This technology has revolutionized the field of implant dentistry by enabling the production of precise and accurate restorations utilizing an industrial manufacturing process, allowing for optimal fit and minimal need for manual adjustment which could reduce treatment time and enhance patient satisfaction [8–10].

Although numerous studies have investigated the mechanical properties of provisional CAD/CAM restorations [11–15], few studies have focused on the biological response of these materials, especially in the context of provisional implant restorations [16, 17]. Provisional restorations are close contact with gingival tissue, hence ensuring their biocompatibility and promoting tissue integration are of paramount importance to prevent any adverse reactions in the adjacent tissues. Evaluating the attachment of human gingival fibroblast (HGF) cells on these restorations becomes critically important as HGFs are primary cells involved in the wound healing process, playing a significant role in maintaining the health and integrity of gingival tissue. By facilitating both biocompatibility and tissue integration, a favorable environment for the natural interaction between the restorations and gingival tissue is established. Furthermore, high bacterial adhesion resistance is crucial for the success of implant treatment, as bacterial colonization can result in plaque formation and an increased risk of periodontal infections. Therefore, further investigation into the biological response of CAD/CAM provisional restorations is necessary to ensure their safety and efficacy in clinical practice.

To address this research gap, this study aims to comprehensively assess the biocompatibility and HGF cell attachment of a range of provisional implant restorations including those using CAD/CAM technology through milling or 3-D printing techniques compared to conventional fabrication of PMMA and bis-acryl-based materials on HGF cells and to evaluate their potential for biofilm formation by periodontal pathogenic bacteria. The rationale of this study is to assess whether the use of novel CAD/CAM provisional restorations is a suitable alternative to conventional fabrication, which is the standard practice in implant treatment, taking into account their biologic response in terms of biocompatibility and resistance to bacterial adhesion. This study's results could inform clinical practice and contribute to improved outcomes in implant therapy.

## Methods

### Sample preparation

Four different commercially available materials and fabrication techniques were used in this study, as demonstrated in Table 1.

**Table 1:** Tested samples

Sample	Manufacturer	Composition	Fabrication technique
Unifast Trad (UT)	GC America, IL, USA	Powder: Methyl methacrylate & Ethyl methacrylate copolymer Liquid: Methyl methacrylate, N,N dimethyl-p-toluidine	Conventional fabrication- Hand mixing, chemical polymerization
Protemp 4 (PT)	3 M ESPE, Seefeld, Germany	Bis-GMA and a second functionalized dimethacrylate resin, silanated zirconia–silica and fumed silica fillers	Conventional fabrication -Automixing, chemical polymerization
VIPIblock -PMMA Trilux (VP)	VIPI, São Paulo, Brazil	Methyl Polymethacrylate, Biocompatible Pigments, EDMA, and Fluorescent	CAD/CAM fabrication - milling technique
Nextdent C&B MFH (ND)	NextDent, Soesterberg, Netherlands	Methacrylic oligomer Glycol methacrylate Phosphine oxide	CAD/CAM fabrication -DLP 3-D printing technique

#### Conventional fabrication groups

A cylindrical silicone mold (diameter 10 mm, thickness 2 mm) was prepared. To create Unifast Trad (UT) samples**,** the powder and liquid were mixed in a dappen dish using a mixing spatula according to a recommended manufacturer's ratio (P/L = 0.5 mL/g) for 10 s and poured into the mold. For Protemp 4 (PT), the material was dispensed into the mold directly using a dispensing gun. A celluloid strip was placed over the material and pressed with a glass slide against the mold to remove the excess material and create a flat surface.

#### CAD/CAM fabrication groups

In CAD/CAM fabrication groups, CAD software (TinkerCAD, Autodesk, California, USA) was used to design the samples and STL files then were exported for sample fabrication. In the VIPIblock -PMMA Trilux (VP) group, a PMMA block was milled using a 5-axis milling machine (S2, VFS, Ammerbuch, Germany). In the Nextdent C&B MFH (ND) group, the 3-D printing process strictly followed the manufacturer's protocol. The printing material (Nextdent C&B MFH) was mixed using an LC-3DMixer (Nextdent, 3DSystems, Rock Hill, SC, USA). The printing parameters were set automatically by Sprint software (3DSystems, Rock Hill, SC, USA) with a 50-micron layer thickness. The specimens then were printed using Nextdent 5100 (Nextdent, 3Dsystems, Rock Hill, SC, USA). Following the printing process, all specimens were cleaned in an ultrasonic unit for 5 min, dried, and cured by a post-curing unit (LC-3DPrint box, Nextdent, 3D Systems, Rock Hill, SC, USA) for 30 min.

All samples were 2 mm in thickness and 10 mm in diameter. Each group replicated three samples for each experiment. The samples underwent polishing using silicon carbide paper grit 600 while being cooled with water. The polished surfaces were visually checked for any irregularities and deemed to be smooth. The samples were then cleaned using an ultrasonic cleaner and wiped down with 70% ethanol. Before each experiment, the samples were exposed to UV light for a duration of 30 min.

### Cell viability analysis via MTT assay

HGF-1 (ATCC CRL-2014, Virginia, USA) in the third to sixth passage were cultured in completed Dulbecco’s modified Eagle medium (DMEM( Sigma Chemical Co., St. Louis, Missouri, USA) supplemented with 10% fetal bovine serum (FBS; Invitrogen, Carlsbad, California, USA) and 100 UT/mL penicillin, 100 μg/mL streptomycin, and 2 mmol/L glutamine (Gibco, Grand Island, New York, USA). The cell lines were maintained under the condition of a humidified atmosphere (5%) of CO_2_ at 37 °C until the cells achieve a confluence of 90%. 10,000 cells were cultured on each material placed in 96-well plate. To determine the cellular responses of four different samples, each sample was immersed in a culture medium (DMEM) for 72 h at 37 °C before treating this medium with HGF. The same density of cells treated with completed DMEM was used as a control. After 24 h of incubation, HGF cellular responses were tested for cell viability by using the colorimetric tetrazolium assay (MTT assay). According to the manufacturer’s protocol, 500 μL of diluted MTT solution (0.5 mg/mL) (Sigma-Aldrich, USA) in serum-free DMEM culture medium was incubated with cell lines grown on each material for 2 h at 37 °C. Then, 100 μL of dimethyl sulfoxide (DMSO) (LGC, Brazil) was added and incubated on a plate shaker for 15 min at room temperature. After the solubilization of the crystal, 200 μL of solution from each well was transferred into a 96-well plate and determined by a microplate reader (Biotek Instruments, CA, USA) at 570 nm, according to standard cultivation times by using reading at 690 nm as a background. The experiment was replicated three times for average cell viability. The cytotoxicity responses were evaluated as a percentage of the control cell viability. Cell viability was assessed according to the previous studies if cell viability above 90% was deemed non-cytotoxic, while viability between 60 and 90% was considered slightly cytotoxic, viability between 30 and 59% was categorized as moderately cytotoxic, and those below 30% were indicative of severe cytotoxicity [18, 19].

### Cell viability analysis via Calcein-AM Staining and confocal microscopy

HGF-1 (ATCC CRL-2014, Virginia, USA) in the third to sixth passage were cultured in completed Dulbecco’s modified Eagle medium (DMEM) (Sigma Chemical Co., St. Louis, Missouri, USA) supplemented with 10% fetal bovine serum (FBS; Invitrogen, Carlsbad, California, USA) and 100 UT/mL penicillin, 100 μg/mL streptomycin, and 2 mmol/L glutamine (Gibco, Grand Island, New York, USA). The cell lines were maintained under the condition of a humidified atmosphere (5%) of CO_2_ at 37 °C until the cells achieve a confluence of 90%. 10,000 cells were cultured on each material placed in 24-well plate and incubated for 24 h. On the day of imaging, 2 µM Calcein-AM (Molecular Probes, Eugene, OR, USA) was added to the cells and incubated for 60 min. Then, the dye was removed and washed the stained HGF cells by incubation with pH 7.2 phosphate-buffered saline (PBS) (Gibco, Thermo Fisher Scientific, USA) for 15 min to allow for AM ester removal. Live cells growing on the materials was analyzed by confocal microscopy (Stellaris 5, Leica Microsystems, Wetzlar, Germany) to determined mean intensity of the green fluorescent.

### Scanning electron microscope (SEM) analysis

The morphology and cell spreading pattern of HGF cells were assessed by SEM (JEOL, Tokyo, Japan) after 24-h incubation with the specimen. 2% Glutaraldehyde was used for fixing the specimens overnight at 4 °C and dehydrated by incubation with increasing concentrations of ethanol. Before SEM scanning, the specimens were coated with a gold/palladium alloy (Agar Sputter Coater) at a voltage of 5 kV. On the day of imaging analysis, the specimens were assessed by SEM at 100X, 200X, 500X, and 1000 magnification.

### Measurement of surface roughness

The surface roughness of all tested samples prepared in the same shape as used in cell culture test was measured using a stylus surface profilometer (Taylor Hobson, Leicester, UK). Each sample was placed on the machine, allowing the stylus to move across its surface. The transducer converts the movement up and down of the stylus along the surface into a signal, which was then processed by the machine's processor. The results are displayed on the screen as a numerical value and visual profile. To calculate the average surface roughness of each sample, the average value from three different areas on each sample was determined.

### Measurement of HGF cell attachment

Each specimen was placed into each well of 24-well plate. 50,000 cells of HGF were loaded on top of the specimen. Then, 24-well plate was incubated in 5% CO2 incubator for 24 h before the measurement of cell attachment. The attached cells on the specimen were fixed with 2.5% glutaraldehyde for 20 min followed by staining with 0.5% crystal violet in 20% ethyl alcohol for 10 min at room temperature. After all incubations, the stained cells were washed 3 times with phosphate buffered saline before adding 200 μL absolute ethanol. The plate was gently shaken for 15 min at room temperature. Then the extracted solution was transferred into a 96-well plate before the measurement of optical density at 590 nM by a microplate reader (Biotek Instruments, CA, USA).

### Measurement of biofilm formation

For the biofilm formation measurement, *Porphyromonas gingivalis (P. gingivalis)* ATCC 33277 strain 2561, a whole-genome sequenced bacterial type strain isolated from a human gingival sulcus, was utilized. The strain was kept at -80 °C and upon requirement, was thawed and transferred to culture in Anaerobic Basal Agar (ABA, Oxoid, Hants, UK) at 37 °C under anaerobic conditions. After 7 days, the single colony of *P. gingivalis* was moved to further culture in Anaerobic Basal Broth (ABB, Oxoid, Hants, UK) at 37 °C under anaerobic condition for 72 h. Then, *P. gingivalis* in broth culture was transferred to Biofilm Induction Media (3% w/v Todd Hewitt Broth (BD, USA) plus 2.2% w/v ABB) and adjusted to achieved microbial density at 1–2 × 107 CFU/ mL. 50 µL of media containing adjusted microbial density was placed on each material and incubated at 37 °C under anaerobic condition for 24 h. Before the analysis of biofilm formation, the biofilm was stained using BacLight Live/ Death (0.75 µM SYTO 9, BacLightTM, molecular probes, Inc., USA) plus 3 µM Propidium iodide (Miltenyi Biotec, Bergisch Gladbach, Germany) for 15 min in light-protected conditions at room temperature. After incubation, the staining solution was discarded, and the biofilm was washed 3 times by Biofilm Induction Media. Then, the biofilm was fixed by 10% natural formalin for 30 min and mounted by using mounting media (Dako fluorescent, CA, USA). After all preparations had finished, biofilm formation was analyzed by confocal microscopy (Stellaris 5, Leica Microsystems, Wetzlar, Germany) to determined biofilm thickness and intensity of the green fluorescent, which represent live microbial biofilms.

### Detection of DNA of *P. gingivalis* via RT-PCR

*P. gingivalis* kept in -80 °C was thawed and moved to culture in Anaerobic Basal Agar (ABA, Oxoid, Hants, UK) at 37 °C under anaerobic condition. After 7 days, the single colony of *P. gingivalis* was moved to further culture in Anaerobic Basal Broth (ABB, Oxoid, Hants, UK) at 37 °C under anaerobic condition for 72 h. Then, *P. gingivalis* in broth culture was transferred to Biofilm Induction Media (3% w/v Todd Hewitt Broth (BD, USA) plus 2.2% w/v ABB) and adjusted to achieved microbial density at 1 × 107 CFU/ mL. Each material was put in each well of 24 mm disc and incubated in 2 mL of media containing adjusted microbial density at 37 °C under anaerobic condition for 72 h. After 72 h, all media containing bacteria was removed from each well. The materials were washed gently with normal saline. Biofilm form by *P. gingivalis* on top of each material was collected a clean 1.5 mL tube using normal saline as a vehicle. The DNA was extracted by using FavorPrep Bacterial DNA Extraction Kit (Favorgen,Taiwan). The extraction procedure was conducted by following the manufacturer's instruction. Nanodrop was used to quantify nucleic acid concentration. PCR using a *P. gingivalis* primer (‘5GCTTCGAAATACGAAACGTG3’, ‘5TATATCCGTCTGTCGGAACG3’) and KAPA SYBR Fast (Biosystem, MA, USA) were implemented to detect *P. gingivalis* DNA and estimated the amount of *P. gingivalis* biofilm on the materials from the standard curve. The standard curve was generated from known amounts of *P. gingivalis* which was added in serial dilution from 50,000 to 5 CFU. The reactions were carried out in a PCR-Real Time Detection System (Biorad CFX96TM, CA, USA), and the fluorescence was monitored throughout the reaction.

### Statistical analysis

Statistical analysis was carried out using Prism® version 6 (Prism software Inc., San Diego, CA, USA). Data are represented as mean ± SEM. One-way ANOVA and Tukey’s HSD post-hoc test were used to compare the cell viability via MTT assay as well as Calcein-AM assay, surface roughness, HGF cell attachment, biofilm thickness, biofilm mean intensity and estimated *P. gingivalis* cell count from DNA detection among four tested sample groups. *P* ≤ 0.05 was considered statistically significant.

## Results

### Cell viability analysis via MTT assay

**Fig. 1:**
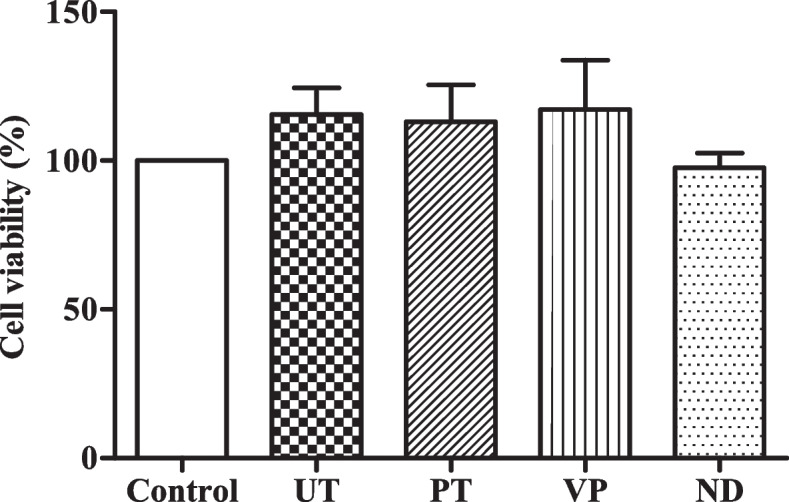
Cell viability of HGF of all tested groups. % HGF viability determined by MTT assay after 24-h incubation with the solution from the 72-h immersion of each specimen. Data are shown as mean ± SEM. Statistical significance was determined using a One-way ANOVA

The study measured the viability and cytotoxic effects of HGFs against both control and tested provisional restorations using MTT assay. The viability percentage of all tested groups was similar to the control as no significant difference was shown between all tested group and control as demonstrated in Fig. 1.

### Cell viability analysis via Calcein-AM staining and confocal microscopy

**Fig. 2:**
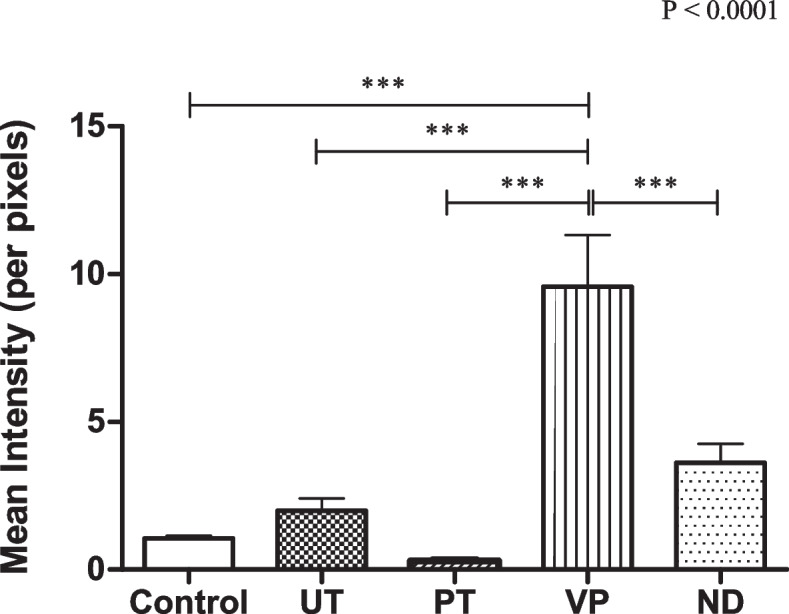
Cell Viability Analysis via Calcein-AM Staining and Confocal Microscopy. Upon analyzing the live cells growing on the materials using confocal microscopy, the mean intensity of the green fluorescent was determined. Data are shown as mean ± SEM. Statistical significance was determined using a One-way ANOVA (*p*-value is shown on the right-hand side) followed by a Tukey’s HSD post-hoc Test, represented by ***P < 0.001

Upon analyzing the live cells growing on the materials using confocal microscopy, the mean intensity of the green fluorescent was determined. The results indicated a significant higher in the mean intensity of VP compared to the control group and other tested groups. However, the mean intensities for UT, PT, and ND showed no significant difference when compared to the control as shown in Fig. 2.

### SEM imaging analyses

**Fig. 3:**
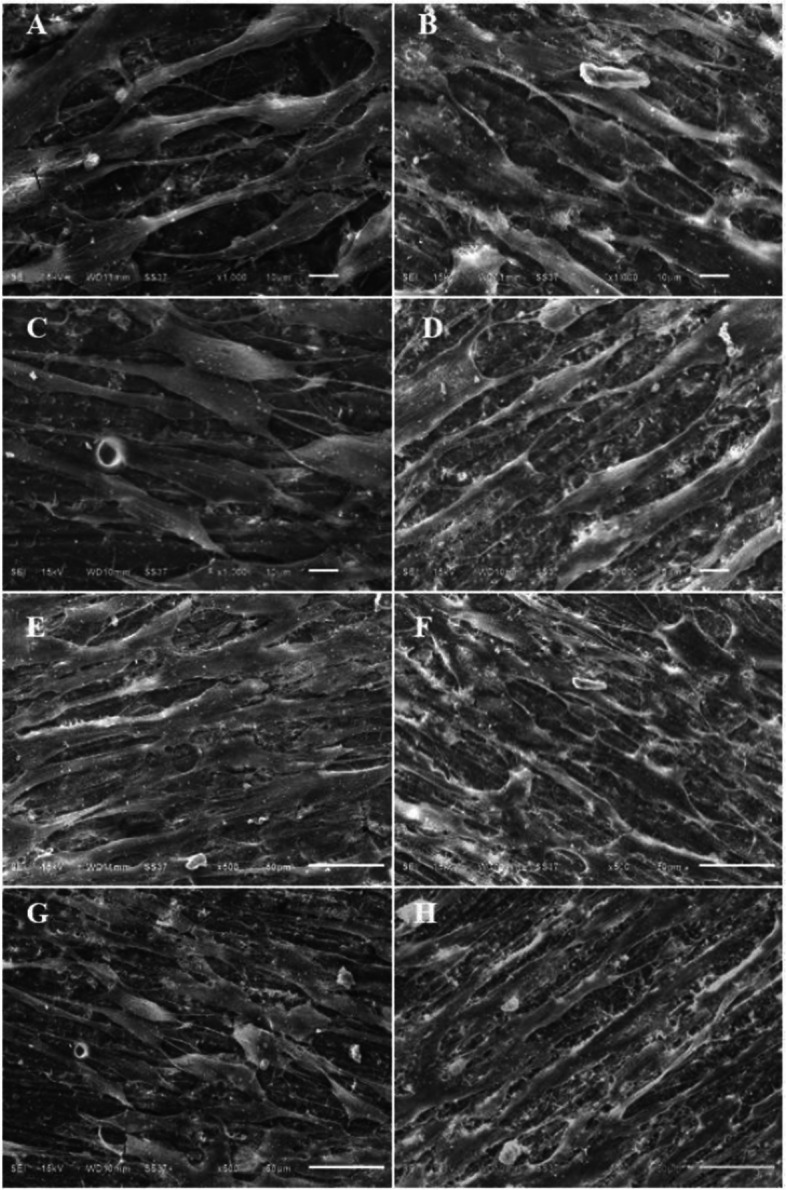
SEM images at magnification 500X and 1000X when HGF were cultured on UT (**A**, **E**), PT (**B**, **F**), VP (**C**, **G**), and ND (**D**, **H**) for 24 h. The scale bars represent 100 μm

**Fig. 4:**
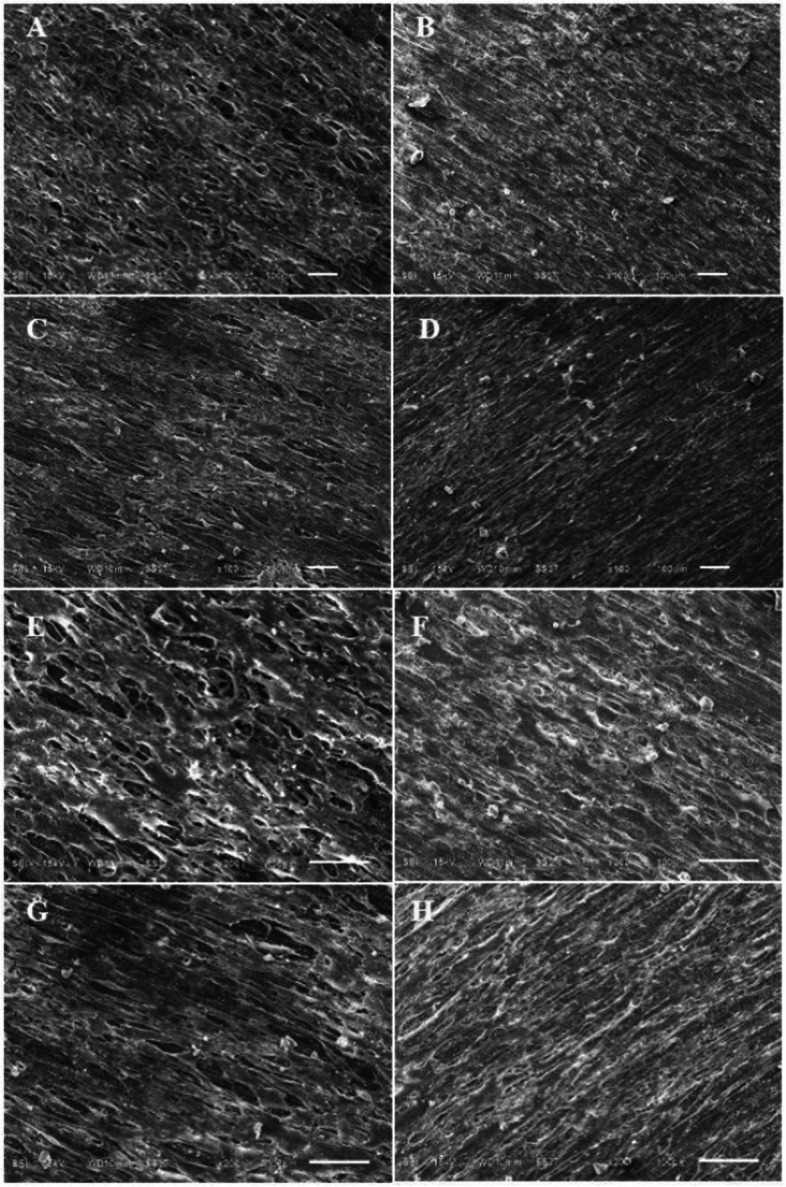
SEM images at magnification 100X and 200X when HGF were cultured on UT (**A**, **E**), PT (**B**, **F**), VP (**C**, **G**), and ND (**D**, **H**) for 24 h. The scale bars represent 100 μm

The imaging analysis by SEM, as presented in Fig. 3, shows both cell morphology and spreading pattern of cell adhesion. The images analyzed at a magnification of 500X and 1000X (Fig. 3) displayed a clear spindle shape with long cytoplasmic elongations after 24-h incubation with all specimens representing good cell viability in all groups. The analyses at magnifications of 100X and 200X (Fig. 4) exhibited cell spreading on the surface of each specimen. The analysis showed generally well-spread and almost confluent HGF on all specimens except the specimen prepared by conventional technique (UT) (Fig. 4A, E), which had less cell adhesion on the material surface than the others.

### Surface roughness

**Fig. 5:**
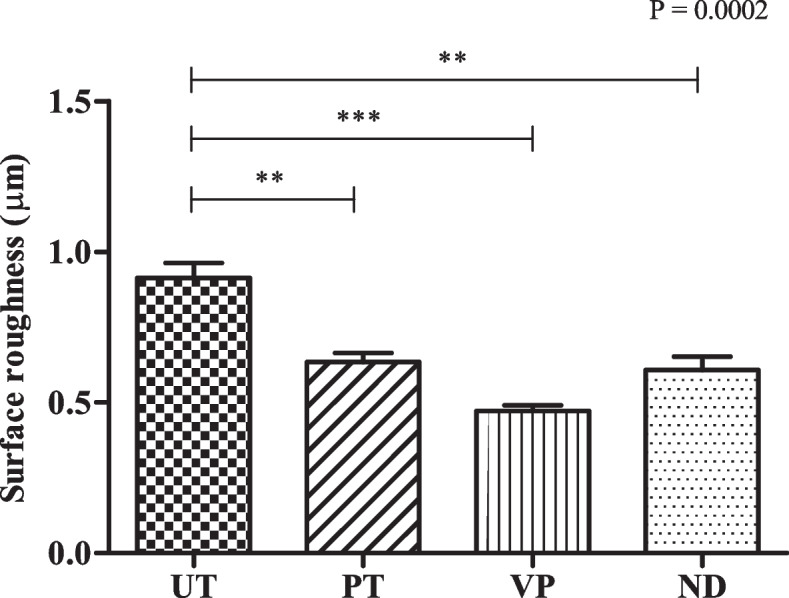
Surface roughness of all tested groups determined by the Stylus Surface Profilometer. Data are shown as mean ± SEM. Statistical significance was determined using a One-way ANOVA (*p*-value is shown on the right-hand side) followed by a Tukey’s HSD post-hoc Test, represented by **P ≤ 0.01, ***P ≤ 0.001

The roughness parameter obtained from the stylus surface profilometer was determined in μm unit (Fig. 5). The VP group provided the smoothest surface, as represented by an average roughness of 0.5 μm. While ND and PT groups showed a similar surface roughness at approximately 0.6 μm, whereas the greatest degree of surface roughness among all groups was produced from UT group which showed an average roughness of 0.9 μm. Statistical analysis revealed a significant difference between the degree of surface roughness of the UT and other groups.

### HGF cell attachment

**Fig. 6:**
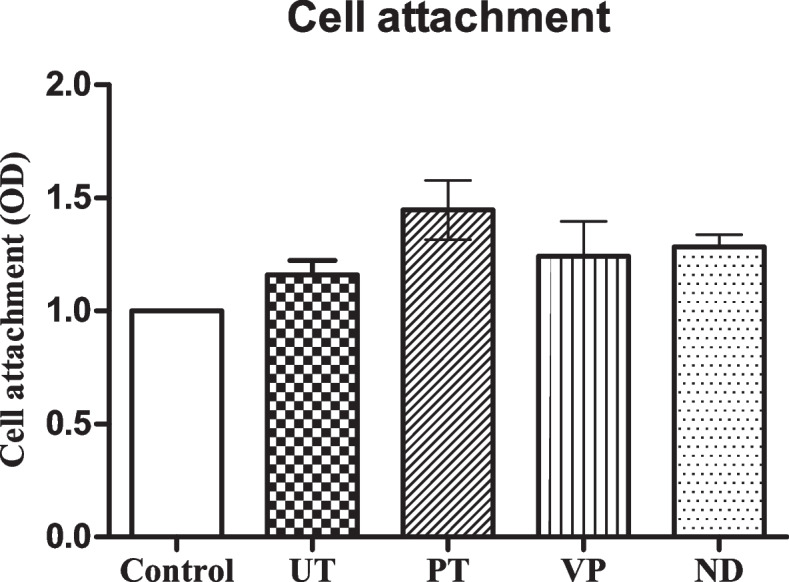
Analysis of HGF Cell Attachment on all tested groups. Data are shown as mean ± SEM. Statistical significance was determined using a One-way ANOVA

The experiment showed greater cell attachment (OD at 590 nm) of HGF cells on the surface of all specimens compared to the control (Fig. 6). Among all specimens, the HGF attachment on the surface of PT specimen was the highest (1.45 ± 0.23 OD), while cell attachment on ND and VP surfaces were 1.29 ± 0.09 and 1.24 ± 0.27 OD respectively. The UT group showed the lowest cell attachment which was 1.16 ± 0.11 OD. However, there was no statistically significant difference of cell attachment between all groups of the specimens.

### Biofilm formation

**Fig. 7:**
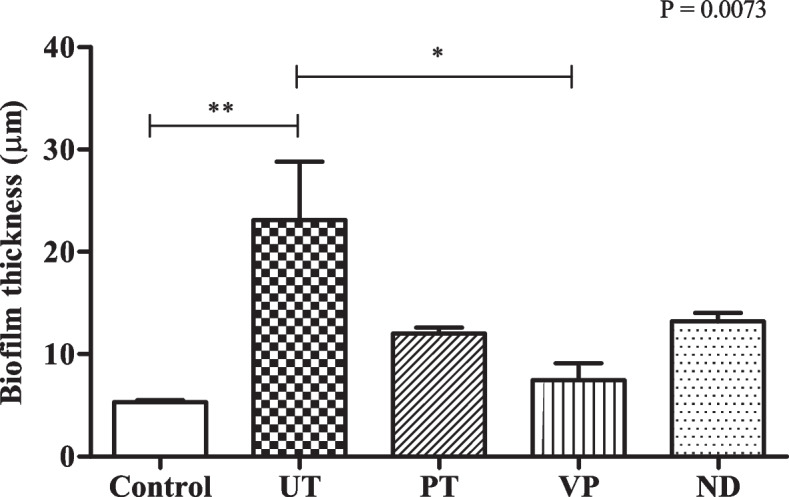
Biofilm formation on all tested groups**.** Thickness of *P. gingivalis* attached to each specimen surface were analyzed by confocal microscopy. Data are shown as mean ± SEM. For all groups *n* = 3. Statistical significance was determined using a One-way ANOVA (*p*-value is shown on the right-hand side) followed by a Tukey’s HSD post-hoc Test, represented by *P ≤ 0.05, **P ≤ 0.01

**Fig. 8:**
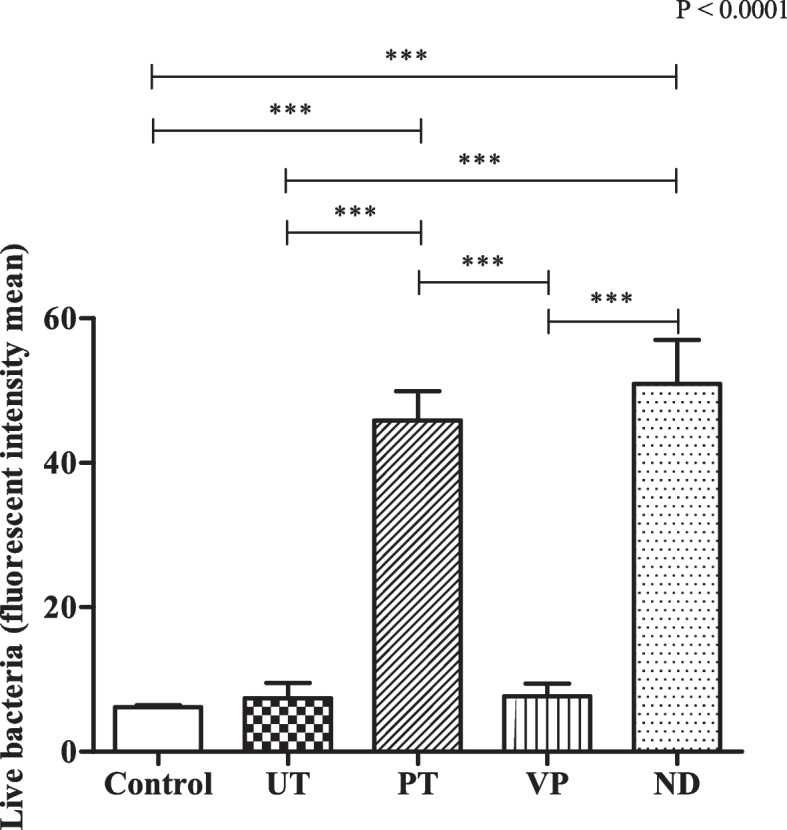
Biofilm formation on all tested groups**.** Mean intensity of *P. gingivalis* attached to each specimen surface were analyzed by confocal microscopy. Data are shown as mean ± SEM. For all groups *n* = 3. Statistical significance was determined using a One-way ANOVA (*p*-value is shown on the right-hand side) followed by a Tukey’s HSD post-hoc Test, represented by ***P ≤ 0.001

**Fig. 9:**
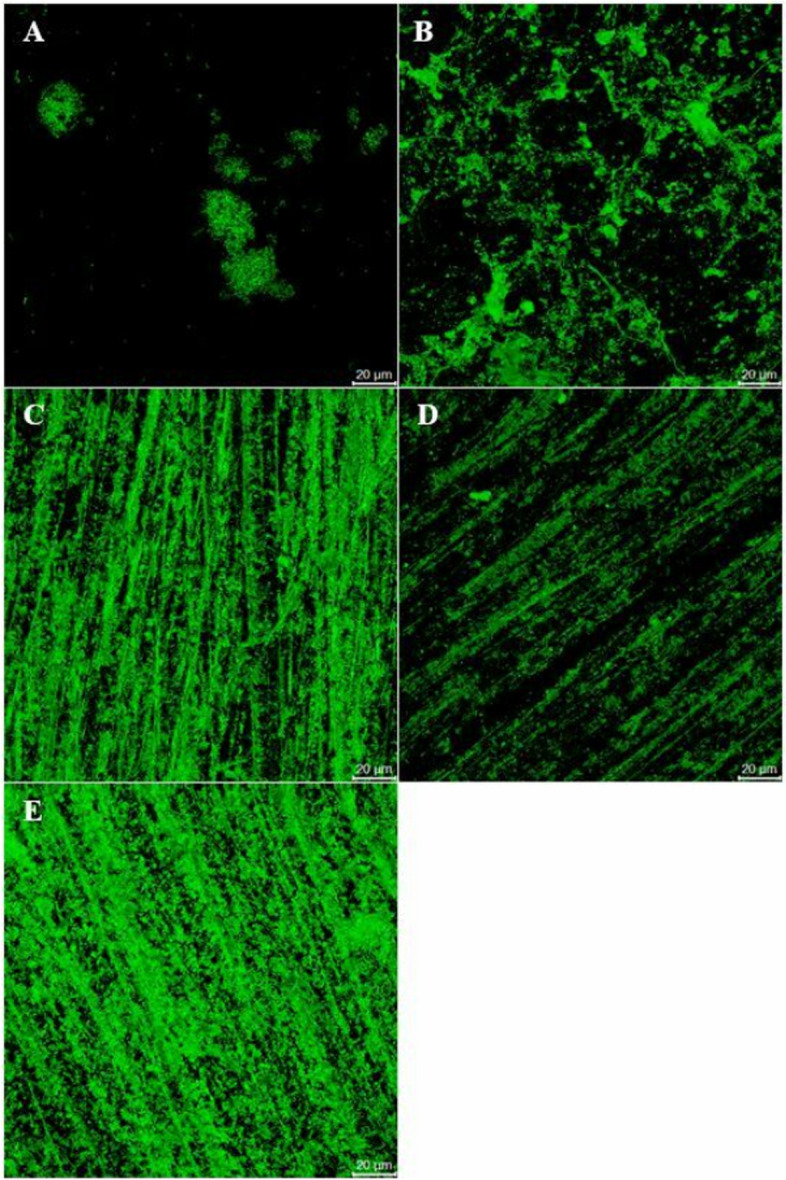
Representative images for biofilm formation on all tested group. The green fluorescent representing live *P. gingivalis* biofilm attached to each specimen surface was captured by confocal microscopy. Imaged analyzed from control (**A**), UT (**B**), PT (**C**), VP (**D**), and ND (**E**) were shown. The scale bars represent 20 μm

The study of biofilm formation analyzed by confocal microscopy revealed that the thickness of *P. gingivalis* attached to the UT surface was highest among all groups of the samples (23.1 ± 9.86 µm) that were statistically significant to the control and VP which had the lowest biofilm thickness (Fig. 7). In terms of *P. gingivalis* intensity mean, ND showed the greatest value compared to the others (50.9 ± 10.55 fluorescent intensity mean) followed by those in the PT (45.9 ± 6.99 fluorescent intensity mean) (Fig. 8). There was a significant difference in *P. gingivalis* intensity mean analyzed from the PT and ND to the other groups. The value of live bacteria analyzed from the PT and ND were significant greater compared to the other groups. Moreover, the different patterns of bacterial formation were observed by confocal microscopy (Fig. 9). Specifically, the bacteria in the PT, ND, and VP groups were arranged in a parallel groove, while the UT group displays circular clusters of bacteria.

### Detection of DNA of *P*. *gingivalis*

**Fig. 10:**
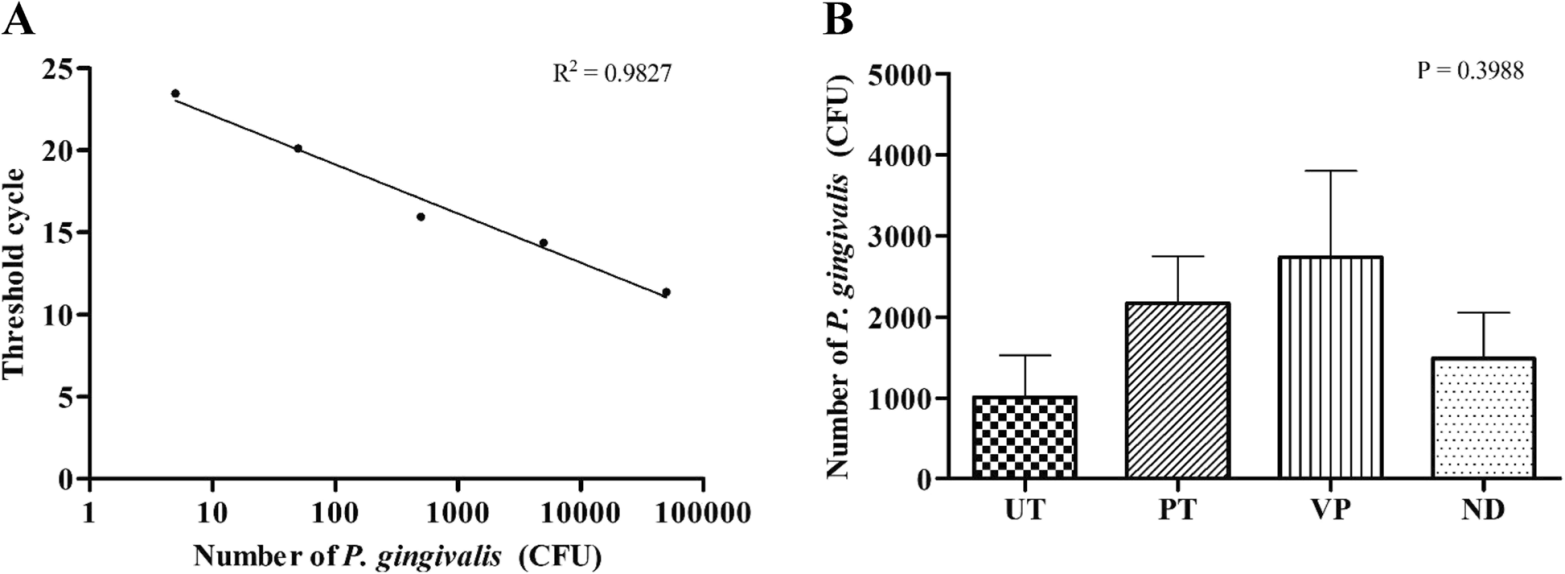
Quantitation of *P*. *gingivalis* DNA extracted from biofilm forming on different type of the specimens by real-time PCR. A standard curve generated from known amount of *P*. *gingivalis* from 50,000 to 5 CFU (R2 = 0.9827) (**A**) was used for a calculation of estimated counted cells of *P*. *gingivalis* forming on each type of specimen (**B**). Data are shown as mean ± SEM. For all groups *n* = 3. Statistical significance was determined using a One-way ANOVA (*p*-value is shown on the right-hand side)

In the experiment focused on the detection of *P*. *gingivalis* DNA, it was observed that *P*. *gingivalis* was present in all tested materials. The bacterial counts ranged from 1,000 to 4,000 CFU across the different materials. However, it's important to note that there was no statistically significant difference in the P. *gingivalis* counts between the various material groups (Fig. 10 A, B).

## Discussions

The complex chemical compositions of novel dental provisional implant restorations fabricated by different techniques have been the subject of various studies [10, 16, 17, 20]. It has been reported that incomplete polymerization of the material and the presence of toxic monomers can negatively affect the biologic response, particularly after surgical procedures such as implant placement where the provisional restoration is in close contact with the surgical site [8, 21]. To evaluate the biocompatibility of these materials, it is essential to assess their ability to maintain cell viability. As a result, the primary objective of this investigation is to evaluate the effect of different materials and manufacturing techniques employed for provisional implant restorations on the biological response of HGF cells.

Assessing the biocompatibility of provisional implant restoration materials with HGF cells is vital for determining their suitability for use in implant therapy, as HGF cells play a crucial role in maintaining the health and stability of the surrounding tissues. In this study, the MTT assay was selected to quantitatively assess the HGF cell viability of tested provisional implant restorations. Both direct and indirect MTT assay methods were used in a previous study on mice gingival fibroblasts to investigate the cell compatibility of these restorations. The findings of that study indicated that conventional fabricated provisional restorative materials were cytotoxic to the cells compared to the control after 24, 48, and 72 h [22]. The use of an indirect MTT assay for assessing the cytotoxicity may be more relevant to the anatomical morphology of the gingival sulcus. This is because the provisional restoration margin is normally placed in a zone of free gingiva [ [23], where indirect exposure is more likely to occur, thus any potential toxic components may be released by the tested sample into the culture medium and can be detected. Specifically, each material was immersed in DMEM for 72 h to allow sufficient time for potentially toxic monomers to be released from the material, and the resultant solution was applied to HGF cells for a 24-h incubation period. The incubation period of 24 h was determined based on previous evidence that revealed the cytotoxicity response of gingival fibroblasts to provisional materials is not time-dependent [22].

Cell toxicity in materials is described as the release of residual monomers or other by-products, which can induce genotoxic effects [24]. The release of residual monomers depends on the chemical composition, degree of conversion, and solvents present under in vivo conditions [10]. Previous studies have indicated that milled PMMA can have a minor cytotoxic effect on HGF cells, and the degree of conversion of prefabricated PMMA blanks may vary across different manufacturers, potentially impacting their cytotoxicity towards HGF cells [20]. However, the results of this experiment indicated that no significant difference was demonstrated between all tested groups and control in cell viability of HGF. This suggests that none of the tested restorations had a negative impact on the gingival fibroblasts, and that these cells could function normally and survive when exposed to the tested restorations. It could be speculated that in this study, all tested specimens were left for a week before testing to simulate the typical indirect fabrication method used in clinical practice. This delay could reduce the amount of unreacted monomer released during chemical polymerization, which in turn lowers the risk of cell toxicity.

The results from the Calcein-AM assay and confocal microscopy analysis provide an understanding of the interactions between HGF cells and various materials. The significant difference in the mean intensity of HGF cells in VP compared to the control group suggests that VP might have unique properties or interactions that affect cell viability differently than UT, PT, and ND. This observation, as depicted in Fig. 2, warrants further investigation into the specific characteristics of VP that contribute to this variance.

While the MTT assay has been used in assessing the viability of HGF cells in different provisional implant restorations The distinct advantage of the Calcein-AM assay is its ability to provide insights into not just the survival, but the real-time functionality of cells. This is crucial because a material might not necessarily be toxic or lethal to cells but could still affect their normal functioning. For instance, while UT, PT, and ND did not show significant differences in cell viability compared to the control, it would be essential to understand if their presence affects cellular functions in any other subtle ways [25].

The use of SEM imaging analysis provided valuable insights into the qualitative analysis of the biocompatibility of the tested restorations in terms of cell morphology and adhesion patterns of HGF cells. The microscopic images taken at 500X and 1000X magnification indicated that the normal morphology of HGF cells was maintained on all tested group. This finding confirms that all tested restoration did not have a harmful effect on the cells. The images captured at 100X and 200X magnification revealed that the HGF cells were well-spread and almost completely covering the surface of all specimens, except for the UT group, which showed some gaps between the cells, suggesting that the cells had not fully formed a continuous layer on the surface. This may be attributed to the inhomogeneous surface characteristic resulting from the manual mixing of the powder and liquid components during fabrication, as opposed to the auto-mixing method used for the other groups [10]. It could be speculated that the inhomogeneous surface of the UT group could hinder cell adhesion on the surface of the restorations since this type of surface could create spaces between the material and the cells, which could reduce the contact area available for cell attachment. Additionally, this inhomogeneous surface could make it difficult for the cells to form stable adhesions, resulting in less cell density on the provisional restoration surface [26].

The secondary objectives of this study covered the evaluation of the amount of biofilm formation and surface roughness of the restorations. The degree of microbial adherence on provisional restorations, with a specific focus on *P. gingivalis*, an anaerobic species associated with periodontal disease [27, 28] was quantified in terms of biofilm thickness and intensity. The surface roughness of these restorations was further evaluated since there have reported the surface roughness was susceptible to the biofilm formation [29]. Through the evaluation of these two parameters, this study attempts to provide valuable insights into the interrelationship between biofilm formation and surface roughness, which have significant implications in the context of dental restorations.

In biofilm formation assay, the bacteria were stained by BacLight Live/Death fluorescent in order to assess the amount of bacteria both live and death. The findings indicated that bacterial growth and survival were not inhibited by any of the tested restorations, as only live bacteria were observed. A closer look at the biofilm formation revealed distinct variations among the tested groups. The UT group, in particular, exhibited the most pronounced biofilm thickness, significantly higher than other groups. This increased biofilm thickness in the UT group aligns with its higher surface roughness value. This finding aligns with previous studies [17, 20, 30]. Furthermore, the intensity of *P*. *gingivalis* varied across the groups, with ND and PT showing notably higher values compared to others. These variations in biofilm formation were further emphasized by the unique bacterial patterns observed under confocal microscopy. For instance, while PT, ND, and VP groups displayed bacteria arranged in parallel grooves, the UT group was characterized by circular bacterial clusters (Fig. 9).

Shifting the focus to the molecular level, the detection of *P*. *gingivalis* DNA across all materials was consistent, with bacterial counts ranging between 1,000 to 4,000 CFU. Yet, it's crucial to highlight that these counts did not exhibit significant differences among the groups. The incorporation of RT-PCR experiments in our study was essential. This technique, known for its high sensitivity and specificity [31], offers a quantitative perspective on *P*. *gingivalis* presence, capturing even trace amounts of bacterial DNA. By complementing our confocal microscopy observations with RT-PCR data, we achieved a more holistic and accurate representation of *P*. *gingivalis* abundance on the tested materials. Such a dual-method approach not only validates our findings but also underscores the intricate interactions of *P*. *gingivalis* with different restorative materials, strengthening the validity of our study.

The roughness observed on the surface of the UT group may be attributed to the presence of air bubbles that were introduced during the manual mixing of the liquid and powder components when filling the external mold [20, 32]. It was expected that UT would show the highest biofilm thickness and intensity due to its highest surface roughness since several studies have shown that rougher surfaces have more bacterial adhesion [33–35]. However, the results indicated that only the thickness of the biofilm was higher compared to other groups, while the biofilm intensity of the UT group was lower than that of the PT and ND groups. It may be speculated that surface roughness and biofilm thickness are correlated, with higher surface roughness potentially leading to greater biofilm thickness. However, there does not appear to be a correlation between surface roughness and biofilm intensity. Therefore, this suggests that surface roughness may not only be a major contributing factor of the amount of viable bacteria in biofilm or biofilm intensity.

Several studies have shown that bacterial attachment is not solely dependent on surface roughness, but also influenced by other factors such as the chemical composition and other surface topographies [17, 36]. Although auto-mixing was employed in the PT group and CAD/CAM was employed in the ND group, which were speculated to be superior to the manual mixing in the UT group, the biofilm intensity in PT and ND was higher than the UT group. It could be explained by the fact that despite all materials being polished with silicon carbide paper grit 600 to simulate the clinical situation, the surface texture of the materials differed as different fabrication techniques were employed for each group. Under a confocal microscope, the surface texture of the PT and ND groups shared a similar specific pattern of parallel grooves leading to a significantly higher biofilm intensity in these two groups compared to others. It is possible to hypothesize that this pattern could promote biofilm formation by providing sites for bacterial attachment and proliferation, resulting in high intensity of biofilm [35, 37].

Several studies have found that the hydrophilicity of the material and presence of some chemical groups that promote or inhibit bacterial adhesion could affect the biofilm formation [29, 38]. From the findings, the biofilm intensity in the UT and VP groups that shared a similar major chemical composition of PMMA was found to be significantly lower than other groups. This suggests that the PMMA may be favorable in preventing bacterial adhesion and resistance.

The results from the HGF cell attachment assay provide an interesting perspective on the characteristics of the tested materials. While surface roughness and texture have been shown to influence bacterial adhesion, However, in this study, despite the variations in surface roughness and fabrication techniques among the groups, there was no significant difference in HGF cell attachment. This uniformity in cell attachment across the groups suggests that the surface properties of the materials, while influential, might not be the sole determinants of cellular behavior. The chemical composition of the material, as well as any surface treatments or coatings, can also influence cell attachment. For instance, certain chemical groups on the material surface can either promote or inhibit protein adsorption, which in turn can influence cell attachment [17, 36].

Interestingly, even though the UT group had the highest surface roughness and was anticipated to exhibit the most cell attachment, the results did not align with this expectation. This might be because HGF cells, being larger than bacteria, may not be as affected by minor surface variations. They could be more responsive to broader topographical features and the material's overall compatibility with living tissue. It's also worth noting that while bacterial adhesion can be harmful, promoting cell attachment is often a desired outcome, especially in the context of dental implants and restorations. Good cell attachment can promote tissue integration, leading to better clinical outcomes. The fact that all tested materials showed good HGF cell attachment, irrespective of their fabrication technique or surface roughness, is a positive indication of their potential suitability for clinical applications.

Selecting the most suitable material and fabrication method in provisional restorations is crucial to minimize any adverse effects in terms of biocompatibility and biofilm formation. The results of the study indicated that the VP group, which contained PMMA and was fabricated using a milling technique, demonstrated a biocompatible response toward the gingival tissue similar to other tested groups. Additionally, the VP group had less *P. gingivalis* biofilm formation.

This study may not fully capture the potential risks or benefits of using these provisional restorations over a prolonged period, as well as the fact that the study was conducted in the in vitro setting that may not fully replicate the complex oral environment and the dynamic interactions between the implant and the surrounding tissues in vivo. Therefore, while the findings of this study provide insights into the biological responses associated with using these restorations, further research conducted in vivo and over a longer period is necessary to fully evaluate their efficacy and safety in clinical settings.

## Conclusions

1. All tested provisional implant restorations were non-toxic and good HGF cell attachment but differed in their quantity of *P. gingivalis* biofilm formation.
2. Surface texture, which can be affected by the type of material and fabrication technique used, may also play a role in the biofilm intensity of *P. gingivalis*.
3. Within the limitation of this study, it seems that the CAD/CAM-fabricated provisional implant restorations using a milling technique (VP) may be the most favorable among the tested groups in terms of biocompatibility and resistance to periodontal-related bacteria adhesion.

## Data Availability

The datasets supporting the conclusions of this article are included within the article.

## Abbreviations

CAD/CAM: Computer-aided design and computer-aided manufacturing
HGF: Human gingival fibroblasts
*P. gingivalis*: *Porphyromonas gingivalis*
PMMA: Polymethylmethacrylate
UT: Unifast Trad
PT: Protemp 4
VP: VIPIblock -PMMA Trilux
ND: Nextdent C&B MFH
DMSO: Dimethyl sulfoxide
SEM: Scanning electron microscope

## References

[1] Burns DR, Beck DA, Nelson SK (2003). A review of selected dental literature on contemporary provisional fixed prosthodontic treatment: report of the Committee on Research in Fixed Prosthodontics of the Academy of Fixed Prosthodontics. J Prosthet Dent.

[2] Hensel F, Koenig A, Doerfler HM, Fuchs F, Rosentritt M, Hahnel S (2021). CAD/CAM resin-based composites for use in long-term temporary fixed dental prostheses. Polymers (Basel)..

[3] Ergün G, Mutlu-Sagesen L, Karaoglu T, Dogan A (2001). Cytotoxicity of provisional crown and bridge restoration materials: an in vitro study. J Oral Sci.

[4] Santosa RE (2007). Provisional restoration options in implant dentistry. Aust Dent J..

[5] Siadat H, Alikhasi M, Beyabanaki E (2017). Interim prosthesis options for dental implants. J Prosthodont.

[6] Zafar MS (2020). Prosthodontic applications of polymethyl methacrylate (pmma): an update. Polymers (Basel).

[7] Singh A, Garg S (2016). Comparative evaluation of flexural strength of provisional crown and bridge materials-an Invitro study. J Clin Diagn Res.

[8] Frasheri I, Aumer K, Keßler A, Miosge N, Folwaczny M (2022). Effects of resin materials dedicated for additive manufacturing of temporary dental restorations on human gingival keratinocytes. J Esthet Restor Dent.

[9] Atria PJ, Bordin D, Marti F, Nayak VV, Conejo J, Benalcázar Jalkh E (2022). 3D-printed resins for provisional dental restorations: Comparison of mechanical and biological properties. J Esthet Restor Dent.

[10] Shim JS, Kim HC, Park SI, Yun HJ, Ryu JJ (2019). Comparison of various implant provisional resin materials for cytotoxicity and attachment to human gingival fibroblasts. Int J Oral Maxillofac Implants.

[11] Jain S, Sayed ME, Shetty M, Alqahtani SM, Al Wadei MHD, Gupta SG (2022). Physical and mechanical properties of 3D-printed provisional crowns and fixed dental prosthesis resins compared to CAD/CAM milled and conventional provisional resins: a systematic review and meta-analysis. Polymers (Basel).

[12] Suralik KM, Sun J, Chen CY, Lee SJ (2020). Effect of fabrication method on fracture strength of provisional implant-supported fixed dental prostheses. Prosthesis.

[13] Reymus M, Fabritius R, Keßler A, Hickel R, Edelhoff D, Stawarczyk B (2020). Fracture load of 3D-printed fixed dental prostheses compared with milled and conventionally fabricated ones: the impact of resin material, build direction, post-curing, and artificial aging-an in vitro study. Clin Oral Investig.

[14] Göncü Başaran E, Ayna E, Vallittu PK, Lassila LV (2011). Load-bearing capacity of handmade and computer-aided design–computer-aided manufacturing-fabricated three-unit fixed dental prostheses of particulate filler composite. Acta Odontol Scand.

[15] Keul C, Martin A, Wimmer T, Roos M, Gernet W, Stawarczyk B (2013). Tensile bond strength of PMMA- and composite-based CAD/CAM materials to luting cements after different conditioning methods. Int J Adhes Adhes.

[16] Mazurek-Popczyk J, Nowicki A, Arkusz K, Pałka Ł, Zimoch-Korzycka A, Baldy-Chudzik K (2022). Evaluation of biofilm formation on acrylic resins used to fabricate dental temporary restorations with the use of 3D printing technology. BMC Oral Health.

[17] Meshni AA, Fageeh HN, Arishi MH, Preethanath RS (2018). Physical Characteristics and bacterial adhesion of computer-aided Design/Computer-aided manufacturing and conventional provisional restorative materials. J Biomater Tissue Eng.

[18] Atay A, Gürdal I, Bozok Çetıntas V, Üşümez A, Cal E (2019). Effects of new generation all-ceramic and provisional materials on fibroblast cells. J Prosthodont.

[19] Sjögren G, Sletten G, Dahl JE (2000). Cytotoxicity of dental alloys, metals, and ceramics assessed by millipore filter, agar overlay, and MTT tests. J Prosthet Dent.

[20] Giti R, Dabiri S, Motamedifar M, Derafshi R (2021). Surface roughness, plaque accumulation, and cytotoxicity of provisional restorative materials fabricated by different methods. PLoS One.

[21] Ulker M, Ulker HE, Zortuk M, Bulbul M, Tuncdemir AR, Bilgin MS (2009). Effects of current provisional restoration materials on the viability of fibroblasts. Eur J Dent.

[22] Campaner M, Takamiya AS, Bitencourt SB, Mazza LC, de Oliveira SHP, Shibayama R (2020). Cytotoxicity and inflammatory response of different types of provisional restorative materials. Arch Oral Biol.

[23] Agustín-Panadero R, Martín-de Llano JJ, Fons-Font A, Carda C (2020). Histological study of human periodontal tissue following biologically oriented preparation technique (BOPT). J Clin Exp Dent.

[24] Pituru SM, Greabu M, Totan A, Imre M, Pantea M, Spinu T (2020). A Review on the Biocompatibility of PMMA-Based Dental Materials for Interim Prosthetic Restorations with a Glimpse into Their Modern Manufacturing Techniques. Materials.

[25] Wang XM, Terasaki PI, Rankin GW, Chia D, Zhong HP, Hardy S (1993). A new microcellular cytotoxicity test based on calcein AM release. Hum Immunol.

[26] Servoli E, Maniglio D, Motta A, Predazzer R, Migliaresi C (2005). Surface properties of silk fibroin films and their interaction with fibroblasts. Macromol Biosci.

[27] Ahn J, Segers S, Hayes RB (2012). Periodontal disease, Porphyromonas gingivalis serum antibody levels and orodigestive cancer mortality. Carcinogenesis.

[28] Zhang Z, Liu D, Liu S, Zhang S, Pan Y (2020). The Role of Porphyromonas gingivalis Outer Membrane Vesicles in Periodontal Disease and Related Systemic Diseases. Front Cell Infect Microbiol.

[29] Aati S, Shrestha B, Fawzy A (2022). Cytotoxicity and antimicrobial efficiency of ZrO(2) nanoparticles reinforced 3D printed resins. Dent Mater.

[30] Simoneti DM, Pereira-Cenci T, Dos Santos MBF (2022). Comparison of material properties and biofilm formation in interim single crowns obtained by 3D printing and conventional methods. J Prosthet Dent.

[31] Lyons SR, Griffen AL, Leys EJ (2000). Quantitative real-time PCR for Porphyromonas gingivalis and total bacteria. J Clin Microbiol.

[32] Köroğlu A, Sahin O, Dede D, Yilmaz B (2016). Effect of different surface treatment methods on the surface roughness and color stability of interim prosthodontic materials. J Prosthet Dent.

[33] Zortuk M, Kesim S, Kaya E, Ozbilge H, Kiliç K, Cölgeçen O (2010). Bacterial adhesion of porphyromonas gingivalis on provisional fixed prosthetic materials. Dent Res J (Isfahan).

[34] Yoda I, Koseki H, Tomita M, Shida T, Horiuchi H, Sakoda H (2014). Effect of surface roughness of biomaterials on Staphylococcus epidermidis adhesion. BMC Microbiol.

[35] Zheng S, Bawazir M, Dhall A, Kim HE, He L, Heo J (2021). Implication of Surface Properties, Bacterial Motility, and Hydrodynamic Conditions on Bacterial Surface Sensing and Their Initial Adhesion. Front Bioeng Biotechnol.

[36] Buergers R, Rosentritt M, Handel G (2007). Bacterial adhesion of Streptococcus mutans to provisional fixed prosthodontic material. J Prosthet Dent.

[37] Li B, Logan BE (2004). Bacterial adhesion to glass and metal-oxide surfaces. Colloids Surf B Biointerfaces.

[38] Yuan C, Wang X, Gao X, Chen F, Liang X, Li D (2016). Effects of surface properties of polymer-based restorative materials on early adhesion of Streptococcus mutans in vitro. J Dent.

